# The Compartmental and Fibrillar Polyhedral Architecture of Fascia: An Assessment of Connective Tissue Anatomy Without Its Abstract Classifications

**DOI:** 10.3390/life15091479

**Published:** 2025-09-20

**Authors:** Graham Scarr

**Affiliations:** Independent Researcher, 60 Edward Street, Stapleford NG9 8FJ, UK; gscarr3@gmail.com

**Keywords:** biotensegrity, extracellular matrix, fascial network, kinematics, musculoskeletal, soft matter, systems, tensegrity

## Abstract

The process of dissection is essential to the study of anatomy, with the variety of colours, shapes, patterns and textures revealing the distinctive features of each anatomical system, but it can also be misleading, because while the body’s constituent ‘parts’ have traditionally been classified according to their appearance, assumed functions and perceived importance, this basic information can be interpreted in different ways. Living organisms are intrinsically indeterminate, which implies that the conclusions arrived at through the *study of anatomy* are not necessarily congruent with the *anatomical reality*, and the abstract classifications of the connective tissues (CTs) are a case in point. This paper highlights a seventeenth-century interpretation of CT anatomy that was pushed aside as the musculoskeletal duality assumed functional dominance and relegated the fascial tissues to mere ancillary roles. In other words, an architectural framework of tensioned fibrous tissues that encompasses a complex body-wide heterarchy of space-filling compartments under compression and reasserts the structural significance of the soft CTs. The problems with orthodox classifications are then discussed alongside a mechano-structural role for the ‘loose’ fibrillar network: a closed-chain kinematic system that guides changes in the relative positions of adjacent compartments and refutes the notion of fascial ‘layers’.

## 1. Introduction

In 1944, Erwin Schrodinger posed the question *What is life?* [1] and examined it in relation to the scientific knowledge available to him at the time, and many others have done the same, but after more than eighty years, how much closer are we to answering that question? The variety of research specialities that feed into such an enquiry seems to be increasing with each passing year, and the enormous amount of information they generate would quickly become overwhelming unless organized in a systematic way.

A random list of animals and plants, for example, would be essentially meaningless on its own, but sorting them into a set of distinct groups—with each member sharing the same defining characteristics of that group—would significantly reduce that diversity to a more manageable level. Indeed, Linnaeus (1758) [2] devised such a system that ranked each organism within a nested multi-level hierarchy of different groups (taxa), which is now well-established in biological classification. *Homo sapiens* appear in the Hominid family group, which is itself contained within a succession of higher-level taxa: Primates, Mammals, Chordates and Animals.

Such a formal ordering of life allows useful comparisons to be made between different categories within the hierarchical tree—both vertically and horizontally—and is naturally very appealing [3,4], with Lamarck (1809/1963, p. 20) [5] describing it as “…an indispensable utility… for enabling us to find our way about among the enormous quantity of different objects that we have to deal with”, and the subject of anatomy has been approached in a similar way.

The human body is hugely complex and intrinsically indeterminate [6], yet a detailed appreciation of anatomy is essential to a proper understanding of normal and abnormal physiology, and as a consequence, it has been simplified in ways that enable that complexity to be more easily managed [7]. Anatomy textbooks thus arrange their contents in a particular order, such as *systems* (bones—joints—muscles—vascular—neural—organs… [8,9,10]) or a more *regional* top-down approach (head—neck—pectoral girdle—upper limb—thorax—abdomen—pelvis—lower limb [11], etc.), with the details of each one then following the system’s ordering. Histology and embryology are then placed on their own and/or within each of the above categories, with the fascial connective tissues appearing as an add-on to the ‘more important’ structures, in other words, abstract, utilitarian classifications that have proved extremely useful to *the study of anatomy*—and its therapeutic applications—but presented misleading impressions of the *anatomical reality* because they are based on outdated traditions and contrived conveniences [7,12,13].

### The Connective Tissues (CTs)

The classification and naming of CTs, for example, has been particularly challenging because they vary considerably in appearance, consistency and composition [13,14,15] and have been “*conveniently* divided into ‘ordinary’ types, which are distributed throughout the body, and special types: namely cartilage and bone” (italics added) and hemolymphoid tissues [10] (p. 44). The ‘ordinary’ (sic) types are then further classified according to their fibre orientation, fluid content and predominant cells, with mucous, serous and basement membranes sitting uneasily within the overall scheme [11]. Williams and Warwick (1980, p. 523) [10] described the fascia as “a term so wide and elastic in usage that it signifies little more than a collection of connective tissue large enough to be described by the naked eye”, a position that has not changed appreciably since [11] (p. 42), while Stecco et al. (2025) [14] proposed a definition of the fascia as a “layered organization of connective tissue” and distinguished a set of *fascial organs* based on their relative positions and associations.

In fact, the body’s constituent ‘parts’ have been generally classified according to their appearance, locality, presumed functions and perceived importance, with the bones assigned to the role of rigid skeleton because of their stiffness and strength [8,11], and muscles as the ‘motors of movement’ becoming firmly embedded with the bones as the *basic structural framework* of the body [16]. It was then inevitable that comparisons with the workings of man-made machines would be made [17,18], with the simplified musculoskeletal duality dominating biomechanical theory because of its analytic convenience [16,19] and the fascial CTs relegated to mere ancillary roles or packing tissue because they cannot be so neatly categorized [11,20], and such a position has become embedded in orthodox thinking. It has influenced every clinician and researcher since the time of Vesalius but unwittingly constrained the study of *architectural* anatomy: the reality of the body’s structural organization without its classificatory abstractions.

## 2. The Architectural Framework

The term *fascia* has a long and varied history that continues to evolve [21,22] and is now appreciated for its involvement in structural stability [12,20], neurovascular/hormonal regulation [23], immunity [24], cancers [25,26], etc., with new definitions and classifications recently introduced [14,27,28]. Stecco et al. (2018) [29] proposed a comprehensive definition of the fascial system as:

“…a three-dimensional continuum of soft, collagen containing, loose and dense fibrous connective issues that permeate the body. It incorporates elements such as adipose tissue, adventitiae and neurovascular sheaths, aponeuroses, deep and superficial fasciae, epineurium, joint capsules, ligaments, membranes, meninges, myofascial expansions, periostea, retinacula, septa, tendons, visceral fasciae, and all the intramuscular and intermuscular connective tissues… The fascial system surrounds, interweaves between, and interpenetrates all organs, muscles, bones and nerve fibers, endowing the body with a functional structure, and providing an environment that enables all body systems to operate in an integrated manner”.

**Figure 1:**
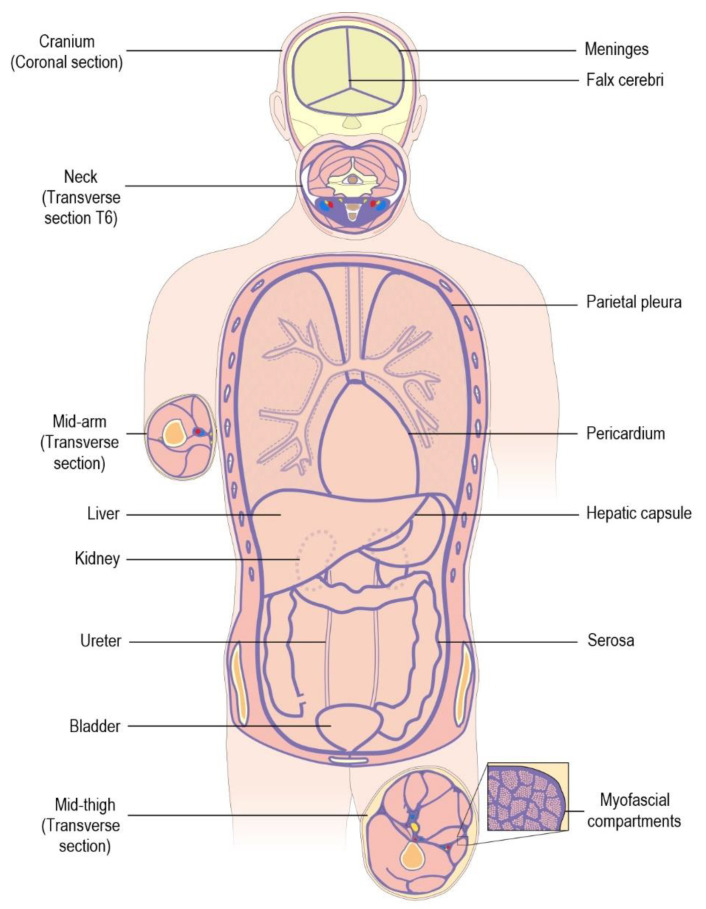
A schematic highlighting the fascia as a ubiquitous fibrous/fluid network enclosing a complex heterarchy of functionally specialized compartments (see text) [5,33,34], and which ultimately surrounds individual parenchymal cells as the extracellular matrix/interstitium (myofascial inset). Reproduced from Scarr et al. (2024), © Elsevier, under Creative Commons BY-NC-ND 4.0 licence [7].

Most importantly, such a description includes a wide range of “macroscopically discernible body parts, the tissues they are composed of and a pervasive soft connective tissue network structure” [22] that unifies many of the inconsistencies found in previous portrayals of the fascia. *However, it must be emphasized* that these ‘parts’ are not discrete entities but structural variants that blend into each other *within a continuum* and have defining characteristics that overlap [15,30,31]. So, while the term *anatomy* relates to “the bodily structure of an organism” [32]—the reality or state of things that actually exists—the dissected evidence is now interpreted in a different way that highlights the compartmental significance of the fascia (Figure 1).

### 2.1. A Heterarchy of Compartments

**Figure 2:**
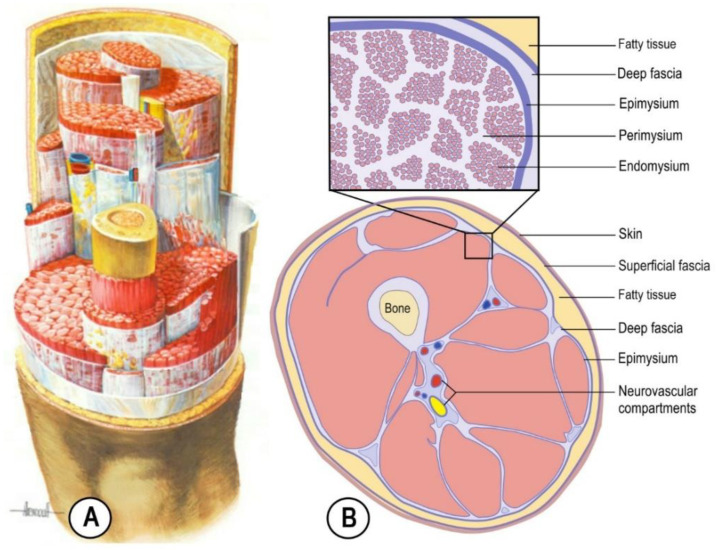
(**A**) The antero-superior view of the left thigh showing transversely sectioned bone and muscular, vascular and neural compartments enclosed within their fascial sheaths; note the smaller perimysial compartments within the sectioned muscles [37]. Reproduced from the World Wide Web [38]: original source unknown. (**B**) The transverse section of the mid-thigh showing the nested hierarchy of fascial compartments (endo/peri/epimysium and deep and superficial fasciae). Reproduced from Scarr et al. (2024), © Elsevier, under Creative Commons BY-NC-ND 4.0 licence [7].

Scarr et al. (2024) [7] described the fascia as “a complex fibrous tissue that contains, connects and encloses multiple sub-systems within the cranium, thoracic/abdominal ‘cavities’ and limbs or in other words, *a* [body-wide] *system of space-enclosing compartments with volume*” [35]. Inside these high-level compartments are smaller fascial compartments encapsulated by the pericardium (heart), parietal pleura (lungs), hepatic capsule (liver), serosa and adventitia (intestines), periosteum (bone), deep [investing] fascia, epimysium (muscles), etc., while even smaller compartments contain local specializations *within* them, e.g., ventricles and peri/endomysium [36,37]. Even blood vessels, nerves, the respiratory airways, the gastrointestinal tract and the ureters can be considered tubular fascial/extracellular matrix (ECM) compartments (Figure 2).

“Each compartment contains smaller compartments that contribute to the bodily framework, with this ‘connective tissue skeleton’ [39] encompassing a complex 3-dimensional (volumetric) heterarchy that extends down to the microscopic level [and fills the entire body], and is capable of supporting itself (c.f. nested ‘Russian dolls’) [30,37] (Figure 2). Each compartment thus carries a dual identity: a regional structural specialization of the fascial/ECM tissues that enclose it, and the more familiar metabolic/functional identity that is provided by the parenchymal ‘power houses’ within: the cardiac cells, hepatic cells, muscle cells, secretory cells, nerve cells and adipose cells, etc.” [7].

“A key role of the fascia… is thus in containing, supporting and facilitating the localized functions of the parenchymal cells within their specialized compartments, which fits with its packing tissue descriptions [20,39] but is now highlighted for its architectural *space-filling contribution* to whole-body anatomy” [7]. This compartmental aspect of CT organization, however, is not a new concept but one that was well-appreciated in the seventeenth century [39].

#### Dissecting the Compartmental ‘Cells’

**Figure 3:**
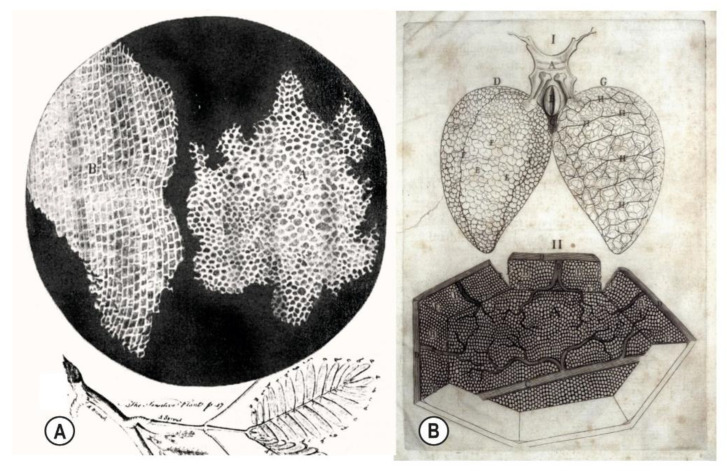
Illustrations from Hooke (1665) showing cork ‘cells’ (**A**) [40] and Malpighi’s (1661) ‘cellular’ compartments within the lungs of a frog (**B**) at macro- (**I**) and micro-scales (**II**) [34].

“One of the definitions of the word *cell* is ‘*a small compartment within a larger structure’* [32] and is a concept introduced by Hooke (1665, Ch. XVIII) [40] in relation to the microscopic structure of plants, and Malpighi (1661, p. 10) [34] when describing the ‘*cellule area interior*’ of the lungs and other organs (Figure 3). Here, the term *cell* had quite different connotations *then* than it does today as it referred to the tissue ‘*spaces*’ and enclosing connective tissue fibres—the ‘cellular’ tissue—but the contents of these ‘cellular spaces’ were poorly understood at the time [39].” [7].

It was a phenomenon that was readily appreciated by von Haller (1747) [33], Lamarck (1809) [5], etc., but gradually faded in significance as attention shifted towards the smaller nucleated (parenchymal) cells in the nineteenth century. Developments in microscopy were now able to elucidate the contents of those compartments, with Schwann’s *nucleated* cell theory attracting attention because of its functional significance [41,42] and the tissue-centric (compartmental) meaning of ‘cellular’ ultimately fading as the parenchymal cells became more prominent [39]; but only the pathological (rather than anatomical) significance of those ‘cellular’ compartments has survived [43].

Lamarck (1809/1963, p. 230) [5], however, had already declared that this “…cellular tissue is the universal matrix of all organization, and that without this tissue no living body could exist”, and the revival of this compartmental aspect of CT anatomy is particularly significant here because it dissolves those awkward classifications (see Section The Connective Tissues (CTs)). It presents an alternative to the way in which structural anatomy is perceived because there are now just two basic categories—tensioned fascial tissues and the compressed compartments they enclose—both of which are dependent on each other and equally important to whole-body architecture, and include an almost infinite variety of local specializations with their own physical and nucleated-cell characteristics [7]. Naturally, the larger, dissectible elements of fascial anatomy have received the most attention [11], but it is their micro-structures that define what they are.

### 2.2. The Extracellular Matrix (ECM)

The fascia essentially consists of an ECM of fibres and a fluid–gel ‘ground substance’—both of which vary in type, composition and relative proportions—along with the different nucleated cell types *that produce and maintain it*, and although usually considered at the microscopic level, the ECM forms the basic structural framework that extends from the smallest to the largest of fascial ‘structures’ [25] and makes standard CT classifications seem rather hollow. It should also be noted that the term *fascia* is considered here synonymous with *soft* CT.

#### 2.2.1. Fluids

The so-called *ground substance* is a complex viscous fluid–gel consisting of water (<15 L [44]), macromolecular proteoglycans and associated glycosaminoglycans (GAGs), and smaller proteoglycans, glycoproteins, polysaccharides, cytokines, growth factors, ions, etc., [25] and contains the fibres and mesenchymal cells within it. It is particularly evident in the fluid-filled (low-fibre density) interstitial ‘spaces’ (1.5–100 μm) that are common *within* the more fibrous ECM [14,45] but also open to the ECM environment in general [46,47]. It is then the intimate association between this fluid/fibrous/gel ECM mix and the tubular capillary and lymphatic compartments that course through it (Section 2.1) that enables the mutual exchange of water, molecules and ions and provides a nutrient resource/‘waste’ disposal/metabolic outlet for cells within both the ECM and parenchymal compartments [44].

These fluid/gel-filled compartments are naturally under compression [45] with the hydrophilic GAGs (particularly hyaluronan [48]), creating an osmotic imbalance that can dramatically increase volume (<1000×) [44] and which is constrained by tensioned collagen fibres and the bulk of adjacent compartments.

#### 2.2.2. Fibres

Collagen, as the most widespread structural protein in the body, occurs in twenty-eight different types, with each one having its own specialized functions [25], and type I fibres (as the most profuse) varying considerably in size, number and orientation. The collagen fibres within fascial sheets, tendons, ligaments and aponeuroses are packed into thick bundles and largely oriented in a direction that aligns with the principal tensional stresses [36]—although their internal organization can be more complicated than this [49]—while the dense bundles of fibres within the reticular dermis, intermuscular sheaths, organ capsules and adventitiae are interweaved in multiple directions and deal with more diffuse tensional stresses [31].

Elastin fibres are thinner than those of collagen type I but significant because of their high compliance and near-perfect elastic recoil, particularly in the skin, lungs, blood vessels, ligamentum flava and ligamentum nuchae. The fibrous fibronectins and laminins also contribute to the organization of the ECM by linking collagen type I with cell surface receptors, and epithelial cells with collagen type IV in basement membranes, respectively [25].

**Figure 4:**
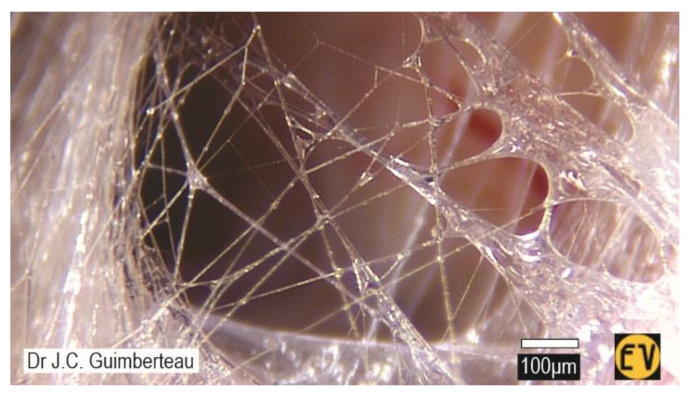
The irregular 3-dimensional organization of fibrils within the ‘loose’ fascia when momentarily lifted from its in situ position during limb surgery (reproduced from Guimberteau and Armstrong, 2015, © Handspring [50]).

However, by far the most general ECM is the ‘loose’ (areolar) fascia with its fluid-filled interstitial ‘spaces’ [14] and fewer, thinner, collagen/elastin fibrils [46] that are randomly organized but enable controlled multidirectional flexibility [30,47] (see Section 3.2.2) (Figure 4). This tissue surrounds and connects individual organs, muscles, vessels and nerves and the interior lobules of organs and perimysial fascicles (i.e., compartments), and supports the visceral and vascular (endothelial) ‘linings’ [45,46], etc. However, traditional classifications that separate ‘loose’ and ‘dense’ CTs are anatomically suspect because (at the microscopic level) they blend into each other, and there is no clear distinction between where one ‘part’ ends and another begins [30].

Adipose is a specialized type of ‘loose’ fascia with an open foam-like structure that consists of vascularized lobular compartments of (nucleated) fatty adipocyte cells contained within fibrous septa and which is particularly prevalent in the mesenteries, peri-viscera and subcutaneous tissues (especially palms and soles) [11]. Similarly, the close association of septal and basement membranes (CT) with epithelial cells in the lining of fluid cavities and compartmental boundaries [25] also suggests local specializations that are otherwise not easily categorized, and at this point, it is necessary to further clarify the meaning of the term ‘cell’ in this context of compartments.

Following from Section Dissecting the Compartmental ‘Cells’, the introduction of Schwann’s nucleated cell theory was significant because it ultimately enabled the contents of those poorly understood compartments [34] to be appreciated for their functional roles—the cardiac cells, hepatic cells, muscle cells, etc.—in other words, parenchymal cells that are considered here as both metabolic entities *and* micro-cellular compartments in the Lamarckian sense [5], with each one surrounded by its own ECM/interstitium. Mesenchymal cells, on the other hand, differ because they are largely contained *within* the ECM/interstitium itself but are also nucleated micro-compartments.

#### 2.2.3. Mesenchymal Cells

There are many different types of these cells within the ECM/fascia, some of which are anatomically localized (e.g., adipocytes, chondrocytes and pericytes [51]) and others that are more mobile (e.g., fibroblasts and fasciacytes), and all are responsive to the changing conditions of normal homeostasis, trauma and pathology [26,52].

Fibroblast (Fb) cells are by far the most abundant and perform a wide diversity of different roles, including the production, maintenance and repair of the ECM, immune regulation [24], etc., but they are not uniform. Fbs express many different phenotypes within a broad functional spectrum (even within the same tissue) that vary according to their interactions with other cells and the local physico-chemical context [53]. They can also transition into more specialized cells with their own functions, such as adipocytes, pericytes [51] and the more contractile *myofibroblasts* that respond to tissue damage and contribute to wound closure, fibrosis and pathological micro-environments [54]. Fasciacytes bear some resemblance to Fbs but are functionally distinct as the primary producers of hyaluronan (Section 2.2.1) [55]. Other cell types include stem cells, telocytes, mast cells, lymphocytes, macrophages, etc., all of which can vary greatly in number and have specialized roles that depend on local conditions, such as those caused by trauma and pathology [11,23].

#### 2.2.4. The Classification Conundrum

It should thus be clear that the highly variable nature of the ECM challenges a precise classification of CTs/fascial tissues (see Section The Connective Tissues (CTs)) because they all blend into each other. The popular use of the term *fascial layers* as a presumed description of anatomical reality [14] is then misleading because it suggests that each tissue ‘layer’ is a distinct entity that can slide against its neighbours during bodily movements [56]. Such ‘layers’, however, are really just *local variants in tissue density and fibre orientation* that are intimately linked through their fibrillar continuities [30], and although these ‘layers’ may *appear* to be different and/or are easily separated during dissection, a mechanical role for this integrating fibrillar network will be presented in the discussion (Section 3.2.2). It is now time to return to the architectural perspective.

### 2.3. Bones and Muscles in Context

This notion of compartments, of course, is not new (see Section Dissecting the Compartmental ‘Cells’) but was gradually pushed aside as attention shifted to its conceptual rival in the nineteenth century—the musculoskeletal duality as the structural framework of the body—but which has now been revived from a perspective that once again appreciates the architectural significance of fascial CTs [7]. So, just like the heart, lungs, liver, intestines, bladder, etc. (Figure 1), bones must now *also* be considered as fascial compartments within this ubiquitous soft CT network that contain their own specialized cells [57,58]. The fibrous material within bone may be mineralized and stiffened, but its complex hierarchical organization [59] also contains, supports and facilitates the functions of the nucleated cells within: the osteoblasts/clasts/cytes and marrow cells [7].

Thus, instead of the hardness and strength of bone being used to justify its traditional role as a rigid framework that “provides support and protection for the body” [11] (p. 88), the bones are now re-interpreted as compartmental specializations of the fascial network that participate in whole-body architecture through their soft periosteal membranes, connected entheses and local mechano-structural characteristics [60] and indicate that cartilage should be considered in a comparable way.

Muscles are then also specialized fascial compartments, with each one defined as a set of contractile parenchymal cells (myocytes) embedded within a complex hierarchy of endomysial, perimysial and epimysial fascia [7], and which serve as the principal generators of fascial tension [61] (Figure 2).

This nested compartmental organization of fascial anatomy (Figure 1, Figure 2 and Figure 3) is thus presented as a “…generalization of the body’s self-support system: a self-stabilizing architectural function that differs significantly from the narrow *musculoskeletal* perspective and has its origins in the embryologic formation of compartmental boundaries [62,63,64]” [7]. It also precludes any authentic classification of CT/fascial anatomy based on its ECM constituents (Section 2.2).

## 3. Discussion

Anatomy is the mainstay of the clinician and inspiration to those who preserve it in its many representations: from life-drawing, images and the written textbook to 3-D modelling, structural analysis [19] and plastination of the entire body [65], its abstract portrayal is a key to understanding who we are. The process of dissection has thus informed those abstractions—and is essential to the study of topographical anatomy [66]—but the information it yields can be interpreted in different ways.

Vesalius (1543/1973, p. 92) [67] referred to the CT as “fleshy membrane” and removed as much of this as possible (during dissection) because it obscured the ‘more important’ bones, muscles, organs, etc., and this remains standard practice [12]. Winslow (1734, I, p. 1) [8] later justified placing the bones at the beginning of the anatomy series (bones–joints–muscles, etc.) with the assertion that “The exact knowledge of the bones is the foundation of all anatomy, because without this, we can never have a just idea of the situation, disposition, connection, and uses of the other parts of the human body”, and as the most complete treatise on descriptive anatomy, his book (1734, I, p. v) was simply expanding on the work of Vesalius and set the trend for classifying anatomy.

### 3.1. The Anatomical Contrivance

The muscle–bone duality then began to assume a prominent position in functional anatomy because of the ease with which it fitted into Borelli’s (1680) machine/lever model of fixed interacting parts [17,68]: a system of weight supporting columns, beams and arches, and muscles that changed their positions in a local piecemeal-like way; or in other words, a contrived duality of rigid bones and contractile muscles that mechanically complemented each other but relegated the fascial tissues to mere supporting roles [7]. Schwann’s (1847) [41] nucleated cell theory, Darwin’s (1862, pp. 283–284) [69] acceptance of the “living machinery” model and developments in mathematics/engineering [70] in the nineteenth century then enabled the musculoskeletal ‘system’ to become firmly established in biomechanical thinking because of its analytic usefulness [19,71] and allowed the compartmental aspect of structural anatomy to be largely forgotten.

The medical need to ‘improve the human condition’ now drives the enormous amount of research that takes place at every level (if only tacitly), and because this is founded in long-standing traditions and practices, it has been able to sidestep the inconvenient approximations, assumptions and unknowns—inherent within its methods—because of this almost sacred orientation [7]. However, as Heidegger (1977, p. 19) [72] noted, “…when man, investigating, observing, ensnares nature as an area of his own conceiving [e.g., the machine model], he has already been claimed by a way of revealing that challenges him to approach nature as an object of research…”. The abstract classifications of CT/fascial anatomy (see Section The Connective Tissues (CTs)) are thus exposed for what they are: the hazily-coupled consequence of traditional mechanistic thinking and the textbook need to contrive some sort of order from the more complex reality.

Indeed, Stecco et al. (2025) [14] recognized that “…many features of the human body are so complex in structure and function that they cross the artificial lines of human imposed categories” but proposed that “the fascial system comprises four anatomical organs: superficial, musculoskeletal (deep), visceral and neural”. However, this subjective division of the fascia into a classification based on “…traditional anatomic definitions of tissues, organs, and systems” [14] is questionable [13] because they are simply local variants of the ECM (Section 2.2) within a now-outdated reductionist ontology [56].

### 3.2. Return to the Compartmental Reality

Hooke (1665, Ch. XVIII) [40], on the other hand, simply described the micro-structure of plants as he saw it—“like honeycomb… bubbles… boxes or cells distinct from one another”, i.e., ‘cellular’ compartments in the original sense (see Section Dissecting the Compartmental ‘Cells’)—while Malpighi (1661, pp. 84–120) [34] observed a similar hierarchical arrangement of nested compartments in dissected fauna, including humans [5,33,73] (Figure 3B), and that was appreciated for its structural significance until well into the 1800s [74]. It is then somewhat ironic that the fading compartmental aspect of anatomy coincided with the rising appreciation of its pathologies by Volkmann (1881) [43], who recognized that the ischaemic contracture of muscles within the limbs was due to an impairment of normal fluid flow and increased interstitial pressure, now known as *Compartment Syndrome* [43], in other words, a patho-functional appreciation of compartmental anatomy that arrived too late to save its architectural significance because of the rapidly-developing research focus on parenchymal cell function [42], the musculoskeletal duality [16] and mathematical analysis [19,70].

#### 3.2.1. Dynamics

Of course, these compartments are not static and isolated but dynamic and intimately associated with each other over a wide range of spatial scales (Figure 1, Figure 2 and Figure 3), and it is inevitable that changes in shape will have an influence on associated tissues. Nucleated cells (mesenchymal and parenchymal) are micro-compartments that change in size, shape, position and number through actin-based force generation [75,76,77] and collectively influence the *mechanical* behaviour of their associated ECM/interstitial/fascial compartments in response to changing physical and chemical stimuli [75,78] and irrespective of their metabolic functions. The formation of compartmental (ECM/fascial) boundaries within the developing embryo is then a natural consequence of this [62,63,64,79] and ultimately leads to the huge variation of compartmental shapes that define an organism’s anatomy: muscles, bones, heart, lungs, intestines, bladder, limbs, neurovascular bundles, etc. (Figure 1 and Figure 2).

A compartment that is expanding in response to the growing parenchymal cells within it and/or changing fluid dynamics will mechanically influence the compartments surrounding it [80,81,82], i.e., it will compress their contents whilst increasing the amount of tension in the containing fascial network—like bubbles within what is essentially a soft-matter foam [83,84] (Figure 2 and Figure 3)—and which, in turn, can limit this expansion through elastic compression and responsive changes in nucleated cell function [64,75,77].

#### 3.2.2. Closed-Chain Kinematics

**Figure 5:**
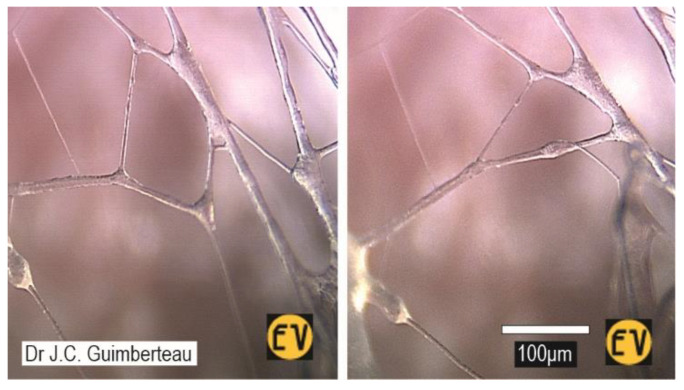
A close-up of the irregular 3-dimensional organization of ‘loose’ fascia (Figure 4), showing how the fibrils spontaneously divide, coalesce and collectively coordinate the response of the polyhedral geometry to changes in tissue tension (reproduced from Guimberteau and Armstrong, 2015, © Handspring) [50].

The structural significance of the tensioned ECM/fascia is then further highlighted by the behaviour of its chaotically organized, three-dimensional fibrillar architecture within the fluidity of the ‘loose’ tissues (Figure 4), as revealed by Guimberteau et al. (2025) during limb surgery [30], which is a prime example of closed kinematic chains in living systems [85,86,87] (Figure 5).

Here, multiple fibrils are coupled into continuous mechanical loops, with each one influencing *all the others* in the system and enabling the controlled transfer and amplification/attenuation of force, speed and kinetic energy. So, while the fibrils are *individually* dividing, coalescing and changing length—in response to the tension/compressional forces imposed on the tissue by bodily movements—their three-dimensional connectivity shows that they are *collectively* coordinating the behaviour of this open polyhedral (closed-chain) geometry in relation to the tissues adjacent to it: the different fascial densifications—or so-called ‘layers’ (Section 2.2.4) [56]—and the compartments they enclose. Conventional descriptions have assumed that this behaviour simply results from the lubricating function of hyaluronan [48]—like the sliding of parts in a machine—but this system of closed-chain kinematics now reveals a mechano-structural role for these fibrillar tissues that are constantly re-configuring within their local ECM environment [88] and guiding changes in the relative positions of associated structures and compartments.

The complexities of the ECM/fascia (Section 2.2) are thus intimately linked with the mechanics underlying its fluid/fibrous/gel/soft-matter dynamics [83,84], with the relationship between this *globally tensioned* fascial network—and the vast interwoven heterarchy of *compressed* compartments within it (Figure 1, Figure 2 and Figure 3)—creating a robust architectural framework that now challenges orthodox descriptions and classifications of structural anatomy (Section 2.1).

### 3.3. The Architectural Context

Tension and compression are inseparable, inextricably linked and cannot exist without each other, and they constitute a mechanical duality that is fundamental to the behaviour of living systems at every level [77,88]. They have thus been appreciated through biotensegrity: a conceptual framework that is founded on a fundamental set of self-organizing principles and includes all the complexities of life, from viruses to vertebrates and molecules to the complete organism [88,89,90,91]. Here, anatomy is considered the physical representation of these hidden forces of tension and compression, with all its ‘parts’ embedded within a unified compartmental architecture [50,89] that enables motion to be controlled from *within the structure itself* [7].

## 4. Conclusions

This paper revives the long-forgotten *compartmental* aspect of CT/fascial anatomy within a unified architectural framework that now fundamentally changes how the human body is perceived. Here, anatomy is no longer considered to be a collection of separate ‘bits’ that operate like a machine—as its orthodox descriptions, classifications and analyses frequently imply (see Section The Connective Tissues (CTs))—but rather a globally tensioned, fluid/fibrous network of ECM/fascial tissues that encloses a complex heterarchical system of regionally specialized compartments under compression, and which extends from the smallest cell to the whole body.

As a consequence, the functionally dominant musculoskeletal duality—which has effectively relegated the fascial tissues to mere ancillary roles for the last two centuries—now becomes conceptually subordinate, with muscles, bones, etc., as compartmental specializations of fascia that participate in whole-body architecture through their associated ECM and local mechano-structural characteristics (Section 2.3). The coupled closed-chain kinematics of the fibrillar polyhedral network within the ‘loose’ fascia (Figure 5) then describes how these inter-linked compartments and related tissues/structures can change position in relation to each other in a controlled way (Section 3.2.2)—irrespective of any neural involvement—and contribute to architectural functionality.

It is thus the fascial network (and all its variations) that defines these anatomical compartments at every level (Section 2.1)—like close-packed bubbles within a foam (Figure 2)—and their reciprocal development within the growing embryo that ultimately determines whole-body morphology [92], in other words, an assessment of CT anatomy that no longer depends on those abstract classifications and enables the reality of the human body to be examined from a more integrated perspective than in the past, and as Lamarck (1809/1963, p. 230) [5] pointed out, “…it is curious to remark how the simplest causes of observed facts are often those which remain the longest unperceived”.

## Data Availability

Not applicable.

## Abbreviations

CT: connective tissue; ECM: extracellular matrix; Fb: fibroblast.
